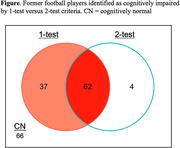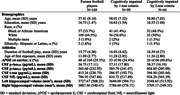# 1‐ versus 2‐test criteria for cognitive impairment and associations with CSF and imaging markers in former American football players

**DOI:** 10.1002/alz.092400

**Published:** 2025-01-03

**Authors:** Monica T. Ly, Caroline Altaras, Yorghos Tripodis, Charles Adler, Laura Balcer, Charles B. Bernick, Henrik Zetterberg, Kaj Blennow, Elaine R. Peskind, Sarah Banks, William B. Barr, Jennifer V. Wethe, Mark W. Bondi, Lisa Delano‐Wood, Robert C. Cantu, Michael J. Coleman, David W. Dodick, Jesse Mez, Joseph N. Palmisano, Brett Martin, Alexander P. Lin, Sylvain Bouix, Jeffrey L. Cummings, Eric M. Reiman, Martha E. Shenton, Robert A. Stern, Michael L. Alosco, DIAGNOSE CTE Research Project

**Affiliations:** ^1^ Boston University Chobanian & Avedisian School of Medicine, Boston, MA USA; ^2^ Boston University Alzheimer’s Disease Research Center, Boston, MA USA; ^3^ Boston University School of Public Health, Boston, MA USA; ^4^ Mayo Clinic, Scottsdale, AZ USA; ^5^ NYU Grossman School of Medicine, New York, NY USA; ^6^ University of Washington, Seattle, WA USA; ^7^ Lou Ruvo Center for Brain Health, Cleveland Clinic, Las Vegas, NV USA; ^8^ UCL Institute of Neurology, London United Kingdom; ^9^ Wisconsin Alzheimer’s Disease Research Center, University of Wisconsin School of Medicine and Public Health, Madison, WI USA; ^10^ Institute of Neuroscience and Physiology, Sahlgrenska Academy, University of Gothenburg, Gothenburg Sweden; ^11^ Hong Kong Center for Neurodegenerative Diseases, Clear Water Bay Hong Kong; ^12^ VA Puget Sound Health Care System, Seattle, WA USA; ^13^ University of Washington School of Medicine, Seattle, WA USA; ^14^ University of California, San Diego, La Jolla, CA USA; ^15^ NYU Langone Health, New York City, NY USA; ^16^ VA San Diego Healthcare System, San Diego, CA USA; ^17^ Brigham and Women’s Hospital, Boston, MA USA; ^18^ Framingham Heart Study, Boston, MA USA; ^19^ Brigham and Women’s Hospital, Boston, MA USA; ^20^ Universite du Quebec, Montreal, QC Canada; ^21^ University of Nevada, Las Vegas, Las Vegas, NV USA; ^22^ Translational Genomics Research Institute, Phoenix, AZ USA; ^23^ University of Arizona, Phoenix, AZ USA; ^24^ Arizona Alzheimer’s Consortium, Phoenix, AZ USA; ^25^ Banner Alzheimer’s Institute, Phoenix, AZ USA; ^26^ Arizona State University, Phoenix, AZ USA

## Abstract

**Background:**

Clinically meaningful cognitive impairment has typically been defined as a single impaired test score, but this approach is prone to false‐positive errors. Examining two test scores at a lower threshold (i.e., using neuropsychological criteria) can improve diagnostic reliability and has shown stronger associations with biomarkers of Alzheimer’s disease. Cognitive impairment in episodic memory and/or executive functioning is a core feature of traumatic encephalopathy syndrome (TES). However, there remains a need to improve the specificity of TES criteria. We applied 1‐ vs. 2‐test criteria for cognitive impairment in former American football players to examine whether 2‐test criteria showed stronger associations with biomarkers of tau, axonal injury, and neurodegeneration.

**Method:**

169 male former American football players from the DIAGNOSE CTE Research Project completed neuropsychological assessment, lumbar puncture, and MRI (see Table). Episodic memory measures were delayed recall from Craft Story, Brief Visuospatial Memory Test, and NAB List Learning. Executive functioning measures were FAS, Stroop Interference, NAB Mazes, and Trails B. Cerebrospinal fluid (CSF) was measured using Lumipulse technology (p‐tau, t‐tau) and an in‐house ELISA (neurofilament light [NfL]). Hippocampal volumes were extracted from structural MRI using Freesurfer 7.1. Cognitive impairment was identified by 1‐test criteria (≥1.5 SD below norms on one test in either domain) and 2‐test criteria (>1 SD below norms on two tests within a domain). Regressions adjusting for age, race, education, and *APOE ε4* status assessed whether meeting 1‐ or 2‐test criteria, separately, predicted log‐transformed CSF p‐tau_181_, p‐tau_231_, t‐tau, and NfL, and hippocampal volumes.

**Result:**

37 of the 99 football players that were impaired by 1‐test criteria did not meet 2‐test criteria, and four uniquely met 2‐test but not 1‐test criteria (see Figure). Cognitive impairment by 2‐test but not 1‐test criteria predicted higher log‐CSF NfL (2‐test: B = 0.24, *p* = .007; 1‐test: B = 0.06, *p* = .53), smaller left hippocampal volume (2‐test: B = ‐195.84, *p* = .01; 1‐test: B = ‐131.52, *p* = .08), and smaller right hippocampal volume (2‐test: B = ‐161.19, *p* = .045; 1‐test: B = ‐134.88, *p* = .08). Neither criterion predicted p‐tau_181_, p‐tau_231_, or t‐tau.

**Conclusion:**

2‐test criteria demonstrated stronger associations between cognitive impairment and markers of axonal injury and neurodegeneration. Incorporating multiple test scores to identify cognitive impairment could improve diagnostic specificity in TES.